# Bifocal or Trifocal (Double‐Level) Bone Transport Using Unilateral Rail System in the Treatment of Large Tibial Defects Caused by Infection: A Retrospective Study

**DOI:** 10.1111/os.12604

**Published:** 2020-01-13

**Authors:** Maimaiaili Yushan, Peng Ren, Abulaiti Abula, Yamuhanmode Alike, Alimujiang Abulaiti, Chuang Ma, Aihemaitijiang Yusufu

**Affiliations:** ^1^ Department of Microrepair and Reconstruction The First Affiliated Hospital of Xinjiang Medical University Urumqi China

**Keywords:** Bifocal bone transport, Distraction osteogenesis, Osteomyelitis, Tibial defect, Trifocal bone transport, Unilateral rail system

## Abstract

**Objective:**

The aim of this study is to assess the clinical results of bifocal or trifocal bone transport using unilateral rail system in the treatment of large tibial defects caused by infection.

**Methods:**

There were a total of 37 eligible patients with an average age of 40.11 ± 10.32 years (range, 18–57 years; 28 males and nine females) with large tibial defects due to infection who were admitted to our hospital from June 2006 to June 2016. Among the patients, 21 underwent bifocal bone transport (BF group), and the remaining 16 were treated with trifocal bone transport (TF group). The demographic data (age, sex, interval duration before bone transport, previous operation time), intraoperative outcomes (size and location of the defect, size of soft tissue defect), postoperative variables (lengthening speed, external fixation index, duration of regenerate consolidation and docking union), postoperative bone and functional outcomes evaluated by Association for the Study and Application of the Method of Ilizarov (ASAMI) scoring system, and postoperative complications evaluated by Paley classification (muscle contraction, axial deviation, delayed consolidation, pin problems, repeated fracture, joint stiffness and others) of the two groups were recorded and compared at a minimum follow‐up of 24 months.

**Results:**

The mean duration of follow‐up after removal of fixator was 29.49 ± 4.34 months (range, 24–38 months). There was no statistically significant difference in the demographic data, intraoperative outcomes including size and location of the defect, size of soft tissue defect, as well as postoperative complications. However, postoperative functional result in the TF group were superior to those in the BF group at a minimum follow‐up of 24 months, and lengthening speed, external fixation index (EFI), duration of regenerate consolidation and docking union were significantly reduced in the TF group when compared with the BF group.

**Conclusions:**

Treatment of large tibial defects caused by infection with trifocal bone transport using unilateral rail system could significantly improve postoperative functional recovery and reduce duration of regenerate consolidation and docking union. The present study provides novel insight for the treatment of large tibial defects caused by infection.

## Introduction

Segmental bone defects represent one of the most challenging conditions to orthopaedic surgeons, especially when they are associated with infection and soft tissue loss due to their long disease course and complex treatment^1^, ^2^. The presence of bony and soft‐tissue defects may be primarily associated with the initial accident in open fractures or, secondarily, after aggressive debridement and resection of infected and necrotic fragments^3^. The aim of the orthopaedic surgeon in such conditions includes radical debridement and resection of any source of obvious or potential infection such as septic bony fragments and infected soft tissue envelop, achieving stable fixation targeting bony union, preserving limb length equality and alignment, and attaining proper function. Numerous procedures exist to bridge tibial segmental bone defects; the specific procedure is determined by the size of the bony defect and the state of the surrounding soft tissues including plate osteosynthesis with cancellous bone grafting, bone grafting with autogenous or allogenic bone grafts, bone shortening and lengthening technique, tibialization of the fibula, vascularized fibular grafts, the Masquelet induced membrane technique, and conventional and modified Ilizarov methods^4^, ^5^, ^6^, ^7^, ^8^, ^9^, ^10^, ^11^, ^12^.

The Ilizarov method, which is used in the treatment of complicated fractures of long bones, was first introduced in 1950 by Gavril Abramovich Ilizarov in the Soviet Union. This revolutionary method for treating fractures, nonunions, deformities and other bone defects involved the use of a circular external fixator. Transport of an osteotomized vascularized bone fragment within the soft tissues to restitute a missing long bone part by distraction osteogenesis (DO) with the Ilizarov method would be an ideal method of bone plasty if not for several known shortcomings and arising problems^9^, ^10^, ^13^, ^14^, ^15^. However, the conventional Ilizarov fixator has its own unique flaws, such as a long period of fixation that leads to significant patient discomfort and stiffness of adjacent joint if physiotherapy is not applied properly, and more importantly, its less applicable characteristics in areas such as the thigh and upper arm. To overcome those drawbacks, the unilateral rail system was introduced which requires less surgical techniques and has greater patient acceptance. Moreover, this device is also more acceptable to the patients because it is less cumbersome. In our previous study, we have concluded that both Ilizarov and Orthofix LRS fixation resolved the bone defects with satisfactory results, and while both led to negative effects on the patient's mental status, the impact of the Orthofix LRS was less severe^16^.

Despite the unique ability to fully induce neo‐osteogenesis, this lengthy technique is limited by undesirable long duration of the consolidation stage and a subsequent increase in the risk of complications^17^. The total duration of distraction osteogenesis can be divided into three phases: the latency phase after osteotomy and application of external fixators; the distraction phase wherein the bone segments proximal and distal to the osteotomy site are separated by gradual and continuous distraction; and the consolidation phase until achievement of sufficient quality^18^. Efforts have been made to decrease the external fixator time in this highly successful technique, particularly to reduce the consolidation phase, which is the longest of the three, by substituting external fixation with internal fixation or multilevel bone transportation. The regeneration of a long bone defect by distraction of one osteotomy site in the bifocal technique takes a long time in the frame, which potentially increases the risk of complications. Borzunov *et al*.^19^ firstly proposed one stage double‐level (trifocal technique) or multilevel bone transport for massive bone defects to shorten distraction time and external fixation time. Their clinical outcomes demonstrated that the duration of distraction could be reduced 2.5 times and fixation improved from 1.3 to 1.9 times with the double level technique compared with the traditional single level technique. However, the trifocal technique needs a more complex assembly of the frame as well as the additional osteotomy. Many authors have reported a significantly shorter treatment time using the trifocal technique in the management of long bone defects^19^, ^20^, but there have been no descriptions of the complications and clinical outcomes compared between bifocal and trifocal bone transport using a unilateral rail system in the treatment of large tibial defects caused by infection.

Based on previous studies and our experience, we hypothesized that trifocal bone transport has advantages over bifocal bone transport regarding lengthening index, regenerate consolidation, docking union, clinical outcomes and overall complications. Therefore, the main purpose of this study are to: (i) explore the therapeutic effect of bifocal and trifocal bone transport using unilateral rail system in the treatment of large tibial defect with or without soft tissue loss; (ii) compare postoperative clinical outcomes and complications between bifocal and trifocal bone transport; and (iii) summarize the exiting limitations and the possible direction for further study. To the best of our knowledge, this is the first clinical study to compare the two techniques using a unilateral rail system in the treatment of large tibial defects caused by infection.

## Materials and Methods

### *The Inclusion and Exclusion Criteria*

*Inclusion criteria*: (i) patients aged 18–65 years old; (ii) tibial defects more than 6 cm and caused by infection with sufficient bone stock near the knee/ankle joint for Schanz screws insertion; (iii) tibial defect treated by bifocal or trifocal bone transport using unilateral rail system; and (iv) a minimum postoperative follow‐up of 24 months with good compliance.

*Exclusion criteria*: (i) tibial defects less than 6 cm and caused by trauma without infection, excision of tumors, congenital defect, limb with vascular insufficiency; (ii) surgical area with poor skin conditions or skin diseases; (iii) severe osteoporosis; (iv) comorbid severe cardiovascular and cerebrovascular diseases, mental illness, and abnormal liver and kidney function; (v) pregnant or lactating women; (vi) autoimmune diseases and blood disorders; and (vii) patients with poor compliance and loss of follow‐up.

### *Included Patients*

In total, 55 patients underwent bone transport technique using unilateral rail system in the treatment of large infective tibial defects. Of these, 18 patients were unavailable for follow‐up (the patients could not be contacted, or the patients were reluctant to take part in the last follow‐up during the outpatient service). The remaining 37 patients were included in this retrospective study, there were 28 males and nine females with mean age of 40.11 ± 10.32 years (range, 18‐57 years). Permission from the Ethics Committee of the First Affiliated Hospital of Xinjiang Medical University was obtained and informed consent was taken from all patients. Data regarding patient's age, sex and side of injury, interval between initial injuries and previous operation time before bone transport were obtained from the patients themselves. All patients had a mean of 2.8 previous surgical treatments. All patients were performed by bifocal (n = 21) or trifocal (n = 16) transport using unilateral rail system (Orthofix limb reconstruction system (LRS)) after radical debridement, with or without spacer filling and soft tissue reconstruction.

Comparison of the demographic preoperative data between BF group and TF group is demonstrated in Table 1. Among the group, 13 patients had substantial soft‐tissue defects, secondary to either the debridement or the initial injury, and were treated with soft‐tissue coverage (six patients treated with free flap, four patients treated with local flap, and three patients covered simultaneously along with bone transport) while Orthofix LRS was applied and before bone transport was initially initiated. We preferred to use 2 g vancomycin or gentamicin per 40 g of cement prepared for spacer filling. Spacer filling was depending on defect size and residual infection consideration evaluated by an experienced surgeon. Direction of bone transport was depending on the location of tibial defect and there were 10 proximal‐to‐distal and 11 distal‐to‐proximal in the BF group and seven proximal‐to‐distal and nine both‐ends‐to‐the‐middle bone transport in TF group.

**Table 1:** Comparison of baseline data between the two groups

Variables	BF group (n = 21)	TF group (n = 16)	*P* values
Age (years)	39.81 ± 9.75	40.05 ± 11.39	0.844
Sex ratio (male/female)	2.50 (15/6)	4.33 (13/3)	0.385
Mean interval duration before BT (month)	16.19 ± 11.02	13.25 ± 7.05	0.359
Mean previous operation time (s)	2.29 ± 1.34	3.13 ± 1.85	0.12
Mean defect size (cm)	7.69 ± 2.32	10.03 ± 3.43	0.018
Injured side (Right/Left)	7/14	6/10	0.532
Localization (Proximal/Middle/Distal)	5/12/7	0/9/7	‐
Culture result (SA/MRSA/PA/EC/Baumanii)	10/7/4/0/0	5/5/3/2/1	‐

BT, bone transport; EC, *Enterobacter cloacae*; MRSA, methicillin‐resistant *Staphylococcus aureus*; PA, *Pseudomonas aeruginosa*; SA, *Staphylococcus aureus*.

### *Surgical Procedure*

#### *Anesthesia and Surgical Position*

The patients were placed in a supine position or lateral position if flap transfer is needed on a radiolucent table under continuous general or spinal anesthesia while application of Orthofix LRS (Shanghai CIIC Medical Instrument Co., Ltd) is administered.

#### *Approach and Exposure*

After obtaining satisfactory surgical position, incision was made according to previous surgical scars or consistent with preoperative flap design; otherwise, anterolateral longitudinal incision was performed, cutting and separating subcutaneous tissue to explore the infected bone.

#### *Resection*

A complete removal of hardware, radical debridement of all necrotic and infected bone and soft tissue, and/or implantation of an antibiotic‐impregnated cement spacer to improve stability were performed prior to bone transport. Cortical bleeding, described as the so‐called “paprika sign”, was accepted as an indication of vital osseous tissue^18^. Specimens were taken and sent for bacterial culture and drug susceptibility tests to guide the surgeon for the appropriate postoperative antibiotics.

#### *Placement of Fixator and Reconstruction*

After debridement, the Orthofix LRS (Shanghai CIIC Medical Instrument Co., Ltd) was assembled for all patients. The external fixator was placed on the distal and proximal fragments parallel to their respective joint based on flap transfer fashion otherwise in anteromedial position (i.e., placement of ORLS is on the anteromedial side of the leg but it need to be placed in proper side if the flap transfer is influenced by the placement). Then, sliding clamps were assembled and Schanz screws were inserted perpendicular to the mechanical axis of the tibia proximal and distal to the defect under the image intensifier control and the desired tibial length and alignment was maintained. Following this, a percutaneous minimally‐invasive osteotomy of the tibia was performed according to pre‐selected osteotomy site(s).

### *Postoperative Management*

**Figure 1:**
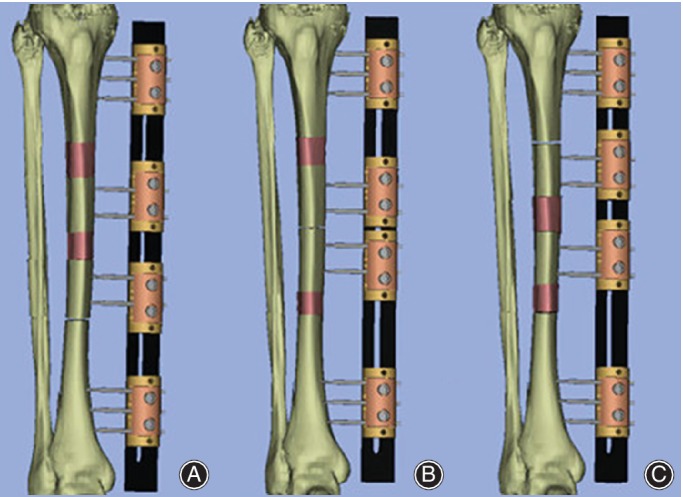
Surgical illustration of trifocal bone transport technique. (A) Trifocal bone transport from proximal to distal using LRS. (B) Trifocal bone transport from both sides using LRS. (C) Trifocal bone transport from distal to proximal using LRS.

Distraction at the osteotomy site(s) was started in both groups on a latency period of 7 to 10 days. Vancomycin or cefuroxime sodium were given intra‐ and postoperatively, and therapy changed based on bacterial culture and drug susceptibility tests results for at least 6 weeks. Passive knee and ankle joint exercises were started at day 1 postoperatively and early full‐weight‐bearing was encouraged. Transport is performed until the docking site is reached. In BF group, fragment was transported at a rate of 0.25 mm four times per day. In TF group, for bone transport in the same direction (proximal to distal), the fragment near the bone defect was transported at a rate of 0.5 mm four times per day, and another fragment far from the defect was transported 0.25 mm four times per day. For converged bone transport, each fragment on both sides of the bone defect proceeded at a rate of 0.25 mm four times per day. The illustration of trifocal bone transport using Orthofix LRS is demonstrated in Fig. 1.

The patients were followed up in the outpatient clinic every 2 weeks, and physical and radiographic examinations were performed to detect and treat common obstacles, problems and complications. When the bridging callus appeared radiologically and limb length equalization was achieved, the frame was dynamized in order to assess the mechanical stability of the regenerated bone and then removed as a daycare procedure. At the time of removal of the external fixator, the leg was protected in a long‐leg cast or cast‐brace for 4 to 6 weeks with the patient using only partial weight‐bearing.

### *Data Collection and Outcome Evaluation*

The raw observation data for the two groups were recorded and compared as follows.

#### *Lengthening Speed*

The lengthening speed was calculated as the amount of lengthening (mm) by the amount of time needed in days for the transported bone segments to reach the docking. In BF group, theoretical lengthening speed was 1 mm per day (0.25 mm four times per day). In TF group, theoretical lengthening speed was 2 mm per day which was calculated by the following rate and rhythm: for bone transport in the same direction (proximal to distal), the fragment near the bone defect was transported at a rate of 0.5 mm four times per day, another fragment far from the defect was transported at a rate of 0.25 mm four times per day, and for converged bone transport, each fragment on both sides of the bone defect proceeded 1 mm per day (0.25 mm four times per day). Lengthening speed was adjusted according to patient's endurance and follow‐up X‐ray evaluation of regeneration.

#### *External Fixation Index*

The external fixation index (EFI) was calculated as a ratio of the number of days the frame was used to the length of regenerated bone (cm). The most common problem in cases of distraction osteogenesis with the bone transport technique in massive bone loss is the long duration of the fixator. The risk of potential complications (both physical and psychological) increased with longer time spent on carrying the external fixator. The time spent in an external fixator (EFI) depends on the length of distraction required and is not free of complications. The removal of the external fixation was based on the following findings: osteogenesis is sufficient in the distraction area, and no deformations were found at the docking site and distraction area when the patient walked during full weight‐bearing activities.

#### *Duration of Regenerate Consolidation and Docking Union*

Duration of regenerate consolidation was defined as total time (days) needed for the appearance of consolidation of at least three cortices on the anteroposterior and lateral radiographs. Duration of docking union was defined as total amount of time (days) required to develop the signs of union (presence of bridging trabeculae on three cortices and absence of pain on dynamization). Radiographs were reviewed monthly to monitor the quality of regenerate during bone transport phase and every 2 months after docking to assess consolidation of the regenerate and healing of docking union. Fischgrund criteria^21^ was used to evaluate consolidation of the regenerate and healing of docking union, when three complete cortices had formed in the regenerate and bone healing was achieved in the docking site according radiographs evaluation. External fixation device cannot be removed until callus mineralization has occurred in the extended segment and docking union has been achieved.

#### *ASAMI Score*

Bone and functional results were evaluated according to the Association for the Study and Application of the Method of Ilizarov (ASAMI) criteria^19^, ^22^. Bone results were evaluated based on union, infection, deformity, and limb length discrepancy and classified as excellent, good, fair, and poor. An excellent result was defined as union, no infection, deformity less than 7°, and leg‐length inequality of less than 2.5 cm; a good result, union plus any two of the other three criteria; a fair result, union plus any one of the other criteria; and a poor result, union but none of the other three criteria, or non‐union or re‐fracture. The functional results were based on five criteria: (i) a noteworthy limp; (ii) stiffness of either the knee or the ankle (a loss of more than 15° of full extension of the knee or of 15° of extension or dorsiflexion of the ankle in comparison with normal contralateral ankle); (iii) soft‐tissue sympathetic dystrophy; (iv) pain that reduced activity or disturbed sleep; (v) inactivity or inability to return to daily activities due to injury and classified as excellent, good, fair and poor (see more details on Table 3). We defined failure of treatment as: (i) recurrent infection with positive cultures from further radiologically guided aspiration or biopsy; (ii) recurrent sinus formation; (iii) further surgery performed for infection; and (iv) any patient requiring long‐term antibiotic treatment for persistent symptoms.

#### *Complications*

Complications were classified according to Paley classification^22^. A problem is defined as a potential expected difficulty that arises during the distraction or fixation period that is fully resolved by the end of the treatment period by nonoperative means. An obstacle is defined as a potential expected difficulty that arises during the distraction or fixation period that is fully resolved by the end of the treatment period by operative means. Complications include any local or systemic intraoperative or perioperative complication, a difficulty during distraction or fixation that remains unresolved at the end of the treatment period, and any early or late posttreatment difficulty. True complications were divided into minor or major. Minor complications were problems that did not require additional surgery and major complications were defined as either obstacles that resolved with additional surgery or true complications that remained unresolved at the end of the treatment period (Table 4).

### *Statistical Analysis*

All statistical analyses were performed using SPSS 21.0 software (IBM software, Chicago, IL, USA). Independent samples *t*‐test was used for parametric quantitative data while the Chi‐square test was used for qualitative data. Fisher's Exact Test was used for qualitative data between both groups. The *P* value of <0.05 indicated a statistically significant difference.

## Results

### *General Results*

A total of 37 patients, 28 male and nine female with an average age of 40.11 ± 10.32 years (range, 18–57 years) were enrolled for our study. The demographic data of recruited patients are described in Table 1. Overall, 21 patients were treated with bifocal bone transport and the other 16 patients were treated with trifocal bone transport. There were no statistically significant differences in age (39.81 ± 9.75 years *vs* 40.05 ± 11.39 years, *P* = 0.844), sex ratio (male/female,15/63 *vs* 13/3, *P* = 0.385), mean internal duration before bone transport(16.19 ± 11.02 month *vs* 13.25 ± 7.05 month, *P* = 0.359), mean previous operation time(s) (2.29 ± 1.34 times *vs* 3.13 ± 1.85 times, *P* = 0.12), injured side (Right/Left, 7/14 *vs* 6/10, *P* = 0.532). There was statistically significant differences regarding the mean defect size (7.69 ± 2.32 cm *vs* 10.03 ± 3.43 cm, *P* = 0.018) between the two groups, and the differences can be explained by selection of bone transport technique (bifocal or trifocal) applied to patients based on both intraoperative defect size and surrounding soft‐tissue condition, the decision on bone transport technique (bifocal or trifocal) was made by the correspondence author (AY). The proportion of bacterial species growth in culture are shown in Table 1. The top three bacterial infections based on culture result are Staphylococcus aureus (40.54%), methicillin‐resistant Staphylococcus aureus (MRSA) (32.43%) and Pseudomonas aeruginosa (18.91%).

### *Postoperative Outcomes*

The mean follow‐up time from removal of the fixator to the time of the last review of 37 patients in this study was 29.49 ± 4.34 months (range, 24–38 months). We compared the postoperative outcome measures of the two groups, and the results are showed in Tables 2, 3, 4.

**Table 2:** Comparison of postoperative data between BF group and TF Group

Variables	BF group (n = 21)	TF group (n = 16)	*P‐*value
Mean lengthening speed (mm/day)	0.79 ± 0.17	1.59 ± 0.26	0.000
Mean EFI (days/cm)	62.21 ± 24.60	32.94 ± 9.21	0.000
Mean duration of regenerate consolidation (days)	202.81 ± 35.22	138.50 ± 31.97	0.000
Mean duration of docking union (days)	299.90 ± 128.26	207.06 ± 40.48	0.005

EFI, external fixation index

**Table 3:** Evaluation of the bone and functional results ASAMI classification

Outcomes	Treatment	Numbers/Percentage					*P‐*value
		excellent	good	fair	poor	failure	
Bone results	BF group	3	16	2	0		0.053
		14.30%	76.20%	9.50%	0.00%		
	TF group	7	6	2	1		
		43.80%	37.50%	12.50%	6.30%		
Functional results	BF group	3	14	4	0	0	0.010
		14.30%	66.70%	19.00%	0.00%	0.00%	
	TF group	9	6	1	0	0	
		56.30%	37.50%	6.30%	0.00%	0.00%	

Criteria

Bone results

Excellent: Union, no infection, deformity <7°, limb length discrepancy (LLD) <2.5 cm.

Good: Union plus any two of the following: absence of infection, deformity <7°, LLD <2.5 cm.

Fair: Union plus any one of the following: absence of infection, deformity <7°, LLD <2.5 cm.

Poor: Nonunion/refracture/union plus infection plus deformity >7° plus LLD >2.5 cm.

Functional results

Excellent: Active, no limp, minimum stiffness (loss of <15°knee extension/<15°ankle dorsiflexion) no reflex sympathetic dystrophy (RSD), insignificant pain.

Good: Active, with one or two of the following: limb, stiffness, RSD, significant pain.

Fair: Active, with three or all of the following: limb, stiffness, RSD, significant pain.

Poor: Inactive (unemployment or inability to return to daily activities because of injury).

Failure: Amputation.

**Table 4:** Complications in 21 bifocal and 16 trifocal tibial bone transport using unilateral fixation system by Paley criteria

Parameter	BF group		TF group				Total
	Problems	Obstacles	Complications	Problems	Obstacles	Complications	
Muscle contraction	3	6	7	6	2	3	27
Axial deviation	8	5	1	5	2	2	23
Delayed consolidation	1	0	0	1	0	0	2
Pin problems	16	5	0	8	4	0	33
Repeat fracture	0	0	0	0	0	1	1
Joint stiffness	0	0	9	0	0	7	16
Other	4	0	0	0	3	0	7
Total	32	16	17	20	11	13	109

#### *Lengthening Speed*

The lengthening speed was 0.79 ± 0.17 mm per day in BF group, while it was 1.59 ± 0.26 mm per day in TF group. It was shown that there was statistical difference (*P* = 0.000) between the two groups, and our results suggest that trifocal bone transport technique possess faster lengthening speed than bifocal bone transport technique which could facilitate early docking contact and potentially reduce EFI by early regenerate consolidation and docking union.

#### *External Fixation Index*

The mean external fixation index (EFI) was 32.94 ± 9.21 days per cm in TF group, and 62.21 ± 24.60 days per cm in BF group. There was significant statistical difference between the two groups (*P* = 0.000). EFI in BF group is almost double than in TF group, and our results indicated that the use of trifocal bone transport technique would significantly impact on the time spent on placement of external fixation and potentially reduce postoperative complications along with psychological burdens.

#### *Duration of Regenerate Consolidation and Docking Union*

The mean duration of regenerate consolidation was 138.50 ± 31.97 days in TF group, and 202.81 ± 35.22 days in BF group. There was conspicuous statistical difference between the two groups (*P* = 0.000). The mean duration of docking union was 207.06 ± 40.48 days in TF group, and 299.90 ± 128.26 days in BF group. There was statistical difference between the two groups (*P* = 0.005). Both regenerate consolidation and docking union are time consuming processes which were also the main reason of lengthy external fixator and could increase potential risk of complications. Our results revealed that faster regenerate consolidation and docking union can be achieved by trifocal bone transport technique, which may potentially reduce postoperative complications and early removal of external fixator.

#### *ASAMI Score*

**Figure 2:**
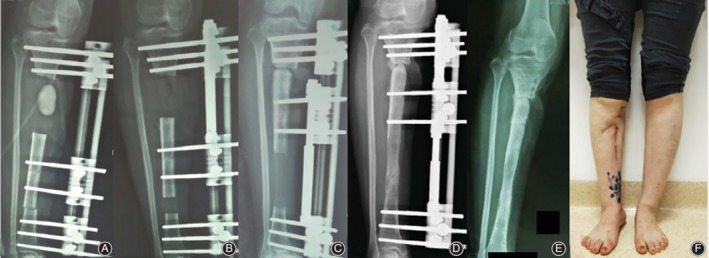
An 32‐year‐old female patient with posttraumatic osteomyelitis of the right tibia was treated at our department with bifocal bone transport from distal to proximal using LRS. (A) An Excision of infected bone and soft tissue with 6 cm defect and filled with cement spacer. (B) Two months after bifocal bone transport using LRS. (C) Docking was reached at 6 months after bone transport with visual regenerate consolidation on X‐ray. (D) Bone transport was completed with good regenerate consolidation and docking union was achieved with bone grafting before dynamization of LRS at 20 months after index surgery. (E) LRS was removed with excellent bone result assessed by ASAMI system. (F) General appearance at last visit on standing position with excellent functional result.

**Figure 3:**
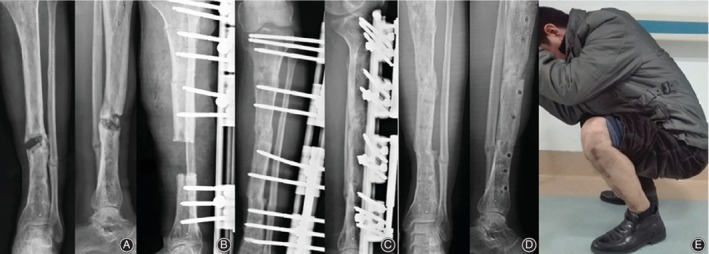
An 42‐year‐old male patient with posttraumatic osteomyelitis the left tibia presented to our department and treated using LRS trifocal bone transport from proximal to distal. (A) Segmental defect of the left tibia caused by infection on X‐ray AP view. (B) Excision of infection bone with 7 cm defect and application of LRS with double level osteotomies for trifocal bone transport. (C) Bone transport was completed with good regenerate consolidation and docking union was achieved and evaluated on AP view of X‐ray at 4 months after index surgery. (D) LRS was removed with excellent bone result shown on AP view of X‐ray at 6 months after operation. (E) Functional recovery at last visit on squatting position at 34 months.

**Figure 4:**
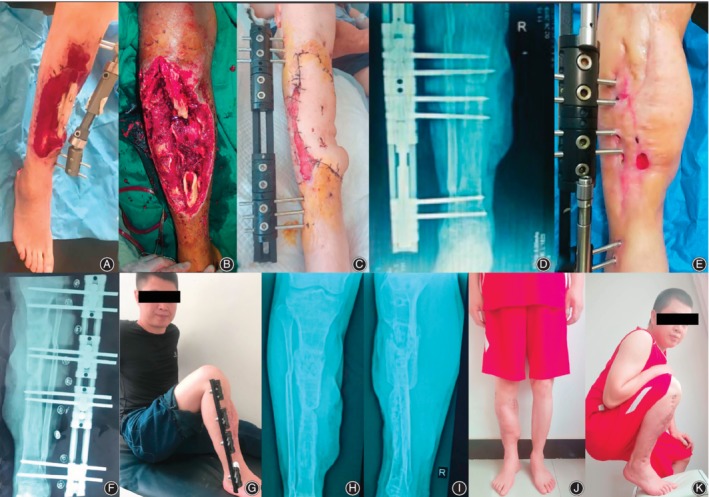
An 38‐year‐old male patient with posttraumatic tibial defect associated with soft tissue defect treated with radial debridemnt, followed by free latissimus dorsi flap transfer and trifocal bone transport using LRS from proximal to distal. (A) Soft tissue defect with tibial shaft exposure after index surgery. (B, C) Removal of devitalized bone and soft tissue by excisional debridement and latissimus dorsi flap is transferred to the coverage of soft tissue defect with OLRS placed on anteriolateral side. (D, E) Trifocal bone transport was completed with successful flap survival in converging direction. (F) Consolidation of regenerates after docking in 3 months. (G, H, I) Functional and radiographic result showed complete consolidation of regenerates on AP view before removal of external fixator. (G, K) Functional recovery of patients showed acceptable range of motion of knee and ankle joint at last visit.

The bone and functional results at last visit (minimum of 24 months) after removal of external fixator is summarized in Table 3, which were evaluated according to Association for the Study and Application of the Method of Ilizarov (ASAMI) classification. There was a statistically significant difference between the two groups in the functional results (excellent/good/fair/poor/failure, 3/14/4/0/0 *vs* 9/6/1/0/0, *P* = 0.010), but not in the bone results (excellent/good/fair/poor/failure, 3/16/2/0/0 *vs* 7/6/2/1/0, *P* = 0.053). Our results indicate that better functional results can be achieved by trifocal bone transport technique by shorter EFI and early removal of external fixator, which could motivate patients to exercise frequently without fixation placement and potentially avoid complications such as muscle contraction and joint stiffness. For more details of the whole procedures in both groups, please refer to Figs 2, 3, 4.

### *Complications*

Complications were classified according to Paley classification. No case encountered joint luxation, vascular or nerve compromise in both groups and the results are shown in Table 4. The complication rate was 3.1 per patient in BF group and 2.8 per patient in TF group (*P* > 0.05). Pin tract infection occurred in 33 cases (30%) which did not interfere with expected clinical results *via* proper management by daily dressing and applying antibiotics based on the result of culture and sensitivity testing. Muscle contraction was encountered in 27 cases (25%) among which 10 cases were recovered by intensive physiotherapy and the remaining 17 were treated by subcutaneous Achilles tendon lengthening or applying the apparatus across the joint and to distract out the contraction. Axial deviation occurred in 23 patients (21%) among which 10 cases were deviated greater than 5°; for recurvature purposes, modification of the apparatus or inserting an additional Schanz screw(s) to pull the bone out of its deviated position was required before the end of the treatment. There were two cases that suffered delayed consolidation which were successfully treated by readjusting lengthening speed or accordion maneuver. Repeated fracture at regenerate was encountered in one case and resolved by applying cast and external fixation until consolidation. Joint stiffness either in knee or ankle was classified as true complication by Paley classification^22^, which occurred in 16 cases (15%) in our study. It was successfully managed by intensive physiotherapy or extending apparatus across the stiffed joint and mobilizing the joint prior to its removal.

## Discussion

The treatment of a large tibial defect caused by infection using bone transport with external fixation has been successful. Among those bone transport approaches, Ilizarov ring fixator has become a gold standard for the treatment of massive tibial bone defects, which could eradicate infection and solve bone and soft tissue defects at the same time^23^, ^24^. However, lengthy external fixation time and overall complications have become the main obstacles to overcome for its extended application. Besides, most patients presented with large tibial defect usually suffered numerous previous surgical interventions and surrounding soft tissues were compromised. How to manage these “characteristic” patients in one stage with shorter treatment course and less complications is an ongoing treatment goal.

Many surgical approaches have been proposed to reduce lengthening index, prevent its potential complications and to accomplish better clinical outcomes. Gupta *et al*.^25^ reported of 14 consecutive infected tibia nonunion patients using simultaneous fixation with a monorail fixator and a locked plate, the mean defect size was 6.4 cm with mean follow‐up of 33.2 months; the result showed the mean external fixator index was 21.2 days per cm and complication rate was 0.5 per patient. Some other surgeons use bone transport over intramedullary nail to avoid wearing an external fixator for a long‐time during defect repair, but the combination cannot accelerate distraction osteogenesis^26^, ^27^. The main drawback of bone transport using a combination of external and internal fixation is additional operation is required, which is difficult for patients to accept considering their numerous previous operations despite its definitive shorter period of external fixator time. Yanlong Zhang *et al*.^28^ treated 16 patients with large post‐traumatic tibial bone defects managed by double‐level bone transport using the Ilizarov technique, and all their patients achieved complete union in both the regenerates and the docking site and eradication of infection. The mean bone transport time was 55.6 ± 23.7 days (range, 30.0–125.0 days). The mean external fixation time was 12.0 ± 3.9 months (range, 5.0–18.0 months), and the mean external fixation index was 1.1 ± 0.3 months/cm (range, 0.8–2.0 months per cm). The bone results were excellent in 10 patients and poor in six patients. The functional results were excellent in 12 patients and good in four patients. In our study, the mean external fixation time was 9.91 ± 2.42 month and the mean EFI was 32.94 ± 9.21 days per cm, the mean duration of regenerate consolidation was 138.50 ± 31.97 days in trifocal bone transport which were comparable to those studies.

Ideally, trifocal bone transport equals faster docking contact which results in early docking union and removal of external fixator once regenerate consolidation are completed. Consolidation of regenerate depends on its length and, more importantly, osteotomy technique and level, and blood supply of transported bone segment. Chaddha *et al*.^29^ reported four cases of twin tibial transport and encountered delayed consolidation of the newly formed regenerate bone from the more distal osteotomy, which is usually carried out in the diaphysis. This can be explained by the exhaustion of the double osteotomized fragment because of the higher incidence of trauma to the nutrient artery. In our study, there were seven cases using proximal‐to‐distal and nine cases using both‐ends‐to‐the‐middle bone transport technique and no delayed consolidation of regenerate have occurred; this can be explained by the use of low energy osteotomy technique and timely adjustment of lengthening speed based on physical and radiological evaluation on regular visit.

The incidence and severity of complications encountered during bone transport have changed dramatically with studies, experience and meticulous prophylactic intervention. The complication rate was 3.1 per patient in our study, among which pin tract infection, muscle contraction and axial deviation were the top three common complications. Pin tract infection is a universal problem which can be successfully treated or even prevented from further development if it is detected in an early stage; regular dressing and improvement of patient's hygiene also plays an important role since it can spread into bone infection if not being managed properly. Muscle contraction was a result of tension generated on the muscle due to distraction and imbalance of strength between flexors and extensors which accounts for 25% of total complications in our study. Muscle contraction of most cases in our study were improved by physiotherapy, or subcutaneous Achilles tendon lengthening or applying the apparatus across the joint to distract out the contraction if needed.

Our present study demonstrates that both bifocal and trifocal bone transport using unilateral rail system led to satisfactory bone and functional results in the treatment of large tibial defects caused by infection. All patients in our series reached bony union with or without bone grafting at the end of the treatment. However, trifocal bone transport led to better functional results but not bony result than bifocal bone transport; this can be explained due to the external fixator not being able to be removed sooner in trifocal group, as well as patients being willing to exercise frequently without fixation placement, which could potentially prevent muscle contraction and joint stiffness. There are many factors that can impact successful distraction osteogenesis based on our experience, such as comprehensive understanding of application of external fixator, prudent patient selection, radial excision of infected bone, timely follow‐up during and after distraction period, early detection of predicted complications and proper surgical or non‐surgical intervention and psychological counseling with in‐charge surgeon during entire treatment course.

The present study had several limitations. First, considering its retrospective nature and relatively small sample size, prudent attitude should be adopted regarding the interpretations of our bone and functional outcomes. Second, longer follow‐up time is necessary to better evaluate the clinical efficacy of the two treatment strategies. Third, further investigations, especially muti‐centered trails with a larger sample size should be conducted to overcome the limitations of our study. Finally, postoperative observational indicators in this study were not comprehensive enough: for example, more accurate quality of life assessments and mental evaluations could be adopted.

### *Conclusion*

Based on our experience on distraction osteogenesis by bifocal or trifocal bone transport using unilateral rail system, we concluded that radical debridement and resection is the most important task in the treatment of tibial defects caused by infection. Preoperative planning is critical for both successful resection and frame mounting. Pin tract infection is a universal problem which can be avoided by regular cleaning. The decision between bifocal or trifocal approaches needs to be decided according to patient, bone quality, defect size, surrounding soft tissue condition, etc. In conclusion, both bifocal and trifocal bone transport using unilateral fixation system can be used successfully to reconstruct large tibial defects caused by infection. It could significantly reduce lengthening index with better functional outcome by trifocal bone transport compared with bifocal bone transport approach.
